# Synergistic effect of thioredoxin and its reductase from *Kluyveromyces marxianus* on enhanced tolerance to multiple lignocellulose-derived inhibitors

**DOI:** 10.1186/s12934-017-0795-5

**Published:** 2017-10-30

**Authors:** Jiaoqi Gao, Wenjie Yuan, Yimin Li, Fengwu Bai, Yu Jiang

**Affiliations:** 1 School of Life Science and Biotechnology, Dalian University of Technology, Dalian, 116024 China; 2 State Key Laboratory of Microbial Metabolism, Shanghai Jiaotong University, Shanghai, 200240 China; 3 Department of Pharmacology and Chemical Biology, University of Pittsburgh, Pittsburgh, PA 15261 USA

**Keywords:** Lignocellulose-derived inhibitors, Tolerance, Thioredoxin, Thioredoxin reductase, Ethanol fermentation, *Kluyveromyces marxianus*

## Abstract

**Background:**

Multiple lignocellulose-derived inhibitors represent great challenges for bioethanol production from lignocellulosic materials. These inhibitors that are related to the levels of intracellular reactive oxidative species (ROS) make oxidoreductases a potential target for an enhanced tolerance in yeasts.

**Results:**

In this study, the thioredoxin and its reductase from *Kluyveromyces marxianus* Y179 was identified, which was subsequently achieved over-expression in *Saccharomyces cerevisiae* 280. In spite of the negative effects by expression of thioredoxin gene (*KmTRX*), the thioredoxin reductase (*KmTrxR*) helped to enhance tolerance to multiple lignocellulose-derived inhibitors, such as formic acid and acetic acid. In particular, compared with each gene expression, the double over-expression of *KmTRX2* and *KmTrxR* achieved a better ethanol fermentative profiles under a mixture of formic acid, acetic acid, and furfural (FAF) with a shorter lag period. At last, the mechanism that improves the tolerance depended on a normal level of intracellular ROS for cell survival under stress.

**Conclusions:**

The synergistic effect of *KmTrxR* and *KmTRX2* provided the potential possibility for ethanol production from lignocellulosic materials, and give a general insight into the possible toxicity mechanisms for further theoretical research.

**Electronic supplementary material:**

The online version of this article (10.1186/s12934-017-0795-5) contains supplementary material, which is available to authorized users.

## Background

More recently, lignocellulose, as the most typical representative of non-grain feedstock, attracts increasing attentions due to low costs and rich sources [1]. Lignocellulose is mainly composed of cellulose, hemicellulose and lignin to form the crystalline microfiber structure, which requires a pretreatment step to produce fermentable monosaccharides for yeasts [2]. Destroying the complex structure of lignocellulose mainly depends on some physicochemical methods like steam explosion, acids or alkali, which may generate inhibitors [3], such as weak acids (formic acid and acetic acid), furan derivatives (furfural and 5-hydroxymethyl furfural), and phenolic compounds (phenol and *O*-methoxyphenol) [4]. In spite of the emerging pretreatment approaches by rot fungi [2], supercritical fluids [5], and ionic liquids [6], the low efficiency makes them to only play a supporting role at the current stage.

Lignocellulose-derived inhibitors are supposed to be inevitable in the hydrolysates, and every single step to reveal resistance mechanism seems to be crucial. Though inhibitors may be removed by detoxification of lignocellulosic hydrolysates [7], loss of sugars and increased costs greatly prevent its wide applications [8]. Therefore, exploring the toxicity of typical inhibitors to cells and its mechanism to achieve excellent strains with enhanced tolerance, is becoming a more vital component of ethanol production from lignocellulosic materials. However, mechanisms of toxicities of these inhibitors in yeasts are very complex and greatly variable depending on strains [9]. Fortunately, inhibitors like acetic acid, furfural and phenol have been reported to be related to the redox state inside cells, inducing reactive oxidative species (ROS) generation [10–12].

Acetic acid generally affects cell metabolism and stabilities of proteins by a drop in intracellular pH and potential, leading to a net negative effect on cell growth and proliferation [13, 14]. Acetic acid diffusing across plasma membrane damages cells by accumulating ROS [10]. Unlike acetic acid, the toxicity of furfural results from the inhibition of glycolytic and fermentative enzymes essential to central metabolic pathways, which reduces cell growth rates eventually [11]. Interestingly, furfural also induces the accumulation of ROS inside cells by lowering activities of intracellular oxidoreductases [15]. Phenolic compounds are supposed to be even more toxic than furfural by generating ROS like peroxides and super oxides inside cells [11]. But beyond that, ionic liquids and hydrogen peroxide have become a new kind of inhibitors as the pretreatment technologies evolve. Hence, oxidoreductases have shown a perfect application in the enhancement of tolerance to multiple inhibitors from pretreatment of lignocellulose.

To reduce levels of intracellular ROS that is induced by multiple lignocellulose-derived inhibitors, the overexpression of oxidoreductases might be a reasonable strategy [16], which may correspondingly contribute to an enhanced tolerance. Actually, expression of some oxidoreductases like mitochondrial cytochrome C oxidase chaperone gene (encoded by *COX20*) improves tolerance to weak acids, especially acetic acid in *S. cerevisiae* [17]. Moreover, an enhancement of intracellular proline concentration by addition of proline or overexpression of a proline synthesis related gene (*PRO1*) decreased the ROS level in yeast cells, which eventually led to an obvious increase in tolerance to both acetic acid and furfural [18]. Beyond that, oxidoreductases like alcohol dehydrogenase *ADH1* [19] and *ADH6* [20], 3-methylglyoxal reductase *GRE2* [21], aldehyde reductase *ARI1* [22] and xylose reductase *XYL1* [23] could be quite effective in enhancing tolerance of yeast cells to inhibitors in lignocellulosic hydrolysates. But, at present, studies have been reported to be applied in increased tolerance to a single inhibitor, few are focused on a mixture of inhibitors above in lignocellulosic hydrolysates [18].

High-throughput sequencing is a powerful tool to gain insight in new genes and their new functions, and helps to reveal the mechanisms of toxicities of lignocellulose-dereived inhibitors [16, 24–26]. Recently, the non-conventional yeast, *Kluyveromyces marxianus*, attracts increasing attentions in the field of industrial biotechnology for its advantages in high-temperature resistance, rapid growth rate and diversity of substrates. With the improvement of technologies of genomics and transcriptomics, more and more new genes or enzymes from *K. marxianus* have been reported for its further theoretical research [27].

Our previous study on transcriptional analysis of *K. marxianus* obtained lots of differentially expressed genes (DEGs) [28], among which oxidoreductases have been proved to be greatly involved in stress tolerance of yeasts [29]. Therefore, an essential defender for ROS in yeasts, thioredoxin (*TRX*) and its reductase (*TrxR*), was further investigated in this study, evaluating the possible applications in stress tolerance under harshest conditions. To test tolerance to multiple inhibitors or stressors, both single expression of *KmTRX* gene, or *KmTrxR* gene, and the synergistic effect of these two genes were achieved in the recombinant *S. cerevisiae*.

## Results

### The potential targets related to stress tolerance in *K. marxianus*

**Fig. 1:**
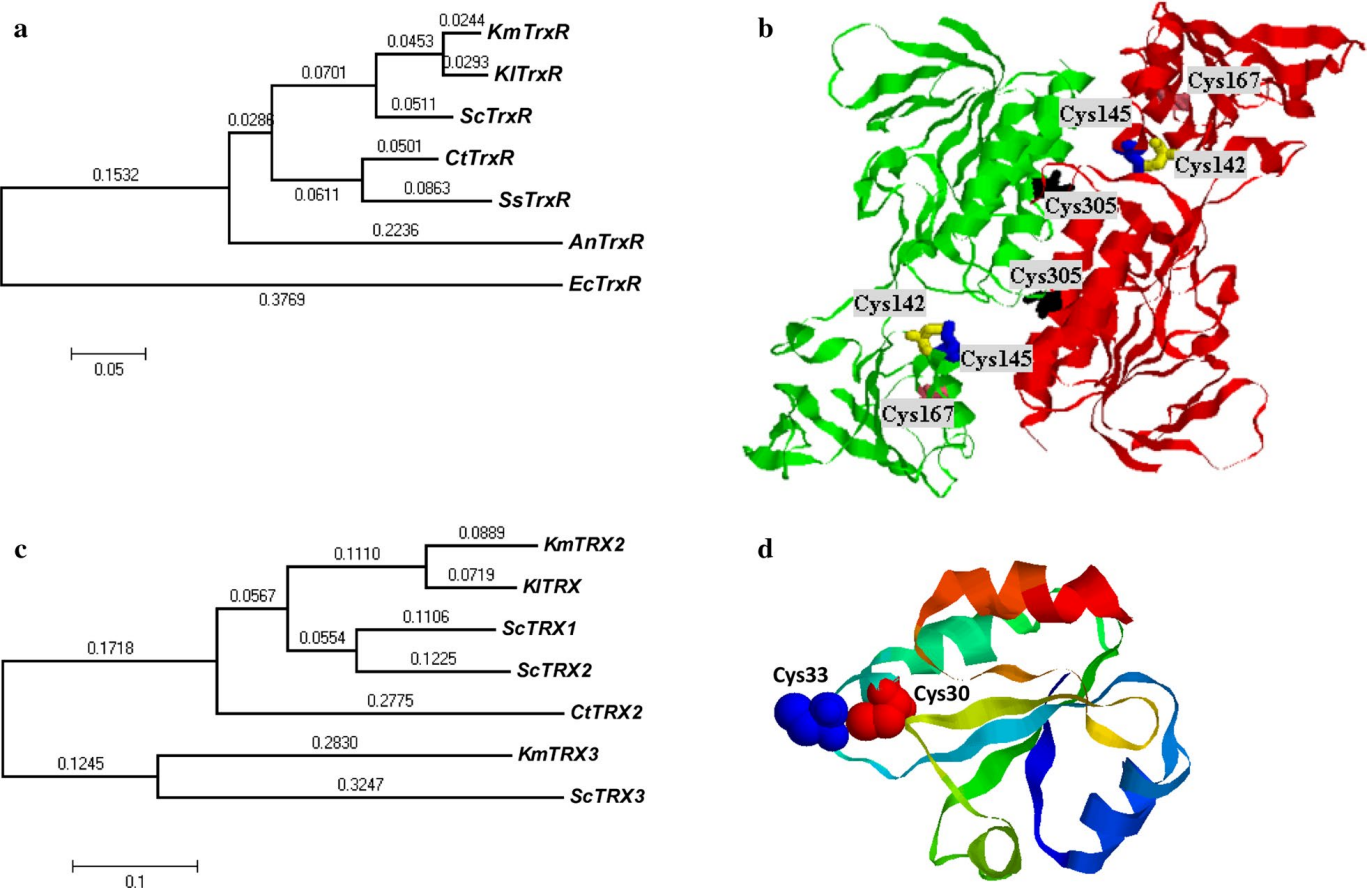
Typical homodimeric proteins, thioredoxin and its reductase, in common yeasts. **a**, **c** Evolutionary trees of thioredoxin reductase and thioredoxin from Y179 and its allied species using *MEGA 4* software. **b**, **d** Predicted 3D structure of thioredoxin reductase and thioredoxin from *K. marxianus*, respectively

The over-expression of thioredoxin family from *K. marxianus*, composed of thioredoxin and its reductase, under harshest conditions (high ROS and ethanol) may be regarded as a potential target for an enhanced tolerance to multiple inhibitors during ethanol fermentation. Thioredoxin reductase belongs to the nucleotide pyridine disulfide oxidoreductase family [30]. The nucleotide sequence of thioredoxin reductase from *K. marxianus* (*KmTrxR*) has an open reading frame of 960 nucleotides, which encodes 319 amino acids. Alignment of *KmTrxR* with other related *TrxR* sequences revealed that active site is highly conserved from bacteria to fungi. *KmTrxR* showed about 50–90% amino acid sequence identities to our selected sequences (Fig. 1a; Additional file 1: Figure S1). Besides, as shown in Fig. 1b, *KmTrxR* is a typical homodimeric protein with the conserved disulfide motifs of Cys142-X-X-Cys145 from a homology modeling of TrxR in *S. cerevisiae* [30]. Two additional cysteine residues (Cys167 and Cys305) close to the active site are identified in *KmTrxR*.

Two kinds of thioredoxins were detected in *K. marxianus*, which is homologous to *TRX2* and *TRX3* in *S. cerevisiae*, respectively (Fig. 1c). *KmTrx2* encodes 104 amino acids, with an approximate molecular weight of 11.2 kDa, and *KmTrx3* encodes 149 amino acids, with an approximate molecular weight of 16.3 kDa. Both thioredoxins from *K. marxianus* possess a redox-active dithiol/disulfide within the conserved active site sequence, which lies in Cys30-Gly-Pro-Cys33 and Cys67-Gly-Pro-Cys70, respectively (Fig. 1d; Additional file 1: Figure S2).

### Functional analysis of single *KmTRX* overexpression in *S. cerevisiae*

**Fig. 2:**
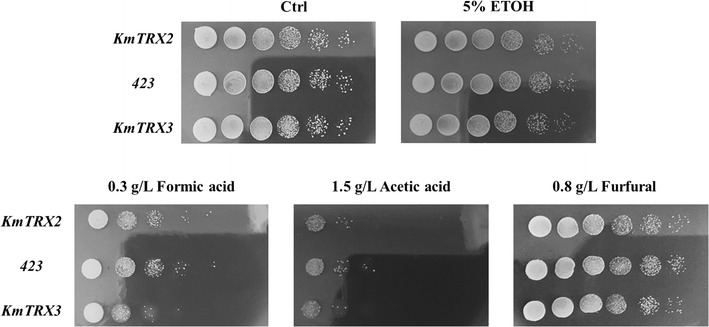
Stress response of single *KmTRX* over-expression to multiple inhibitors by serial dilution assay. Cells in log phase with OD_620_ of 10 were serially diluted to 10^−5^, and then spotted onto SC-His plates containing various inhibitors. Cells were cultivated at 30 °C for 3 days and then photographed

*Saccharomyces cerevisiae* 280 cells overexpressing *KmTRX2* or *KmTRX3* genes were adopted to evaluate the possible functions in an enhanced tolerance to multiple inhibitors (Fig. 2). The growth behavior of overexpressed strain Trx2 and control strain 423 displayed no obvious differences without, or with multiple inhibitors. Unfortunately, inhibitors like formic acid and acetic acid even repressed the growth of strain Trx3. These findings indicated that the single overexpression of gene *KmTRX2*, or *KmTRX3*, contributed little to the increase of stress tolerance in *S. cerevisiae*.

### Functional analysis of single *KmTrxR* overexpression in *S. cerevisiae*

**Fig. 3:**
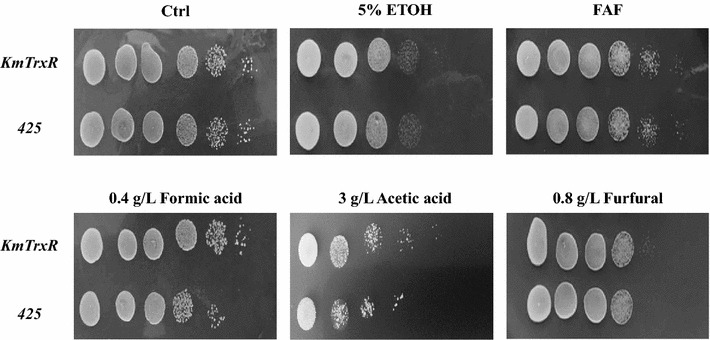
Stress response of *KmTrxR* over-expression to the presence of multiple lignocellulose-derived inhibitors by serial dilution assay. Cells in log phase with OD_620_ of 10 were serially diluted to 10^−5^, and then spotted onto SC-Leu plates containing various inhibitors. Cells were cultivated at 30 or 42 °C for 3 days and then photographed

To test the functions of *KmTrxR* in increasing tolerance to lignocellulose-derived inhibitors, weak acids (formic acid and acetic acid) and furfural were firstly selected as the representative inhibitors in hydrolysates. As shown in Fig. 3, gene *KmTrxR* played a positive role in the enhanced tolerance to formic acid and acetic acid. Over-expression of *KmTrxR* would not affect the cell growth, while the growth of control strain 425 had been greatly repressed on plates with 0.4 g/L of formic acid and 3 g/L of acetic acid, which was 1–2 gradients less than strain TrxR in the serial dilution assay. However, the effect of gene *KmTrxR* in promoting the tolerance to furfural, another typical lignocellulose-derived inhibitor, was not very obvious.

**Fig. 4:**
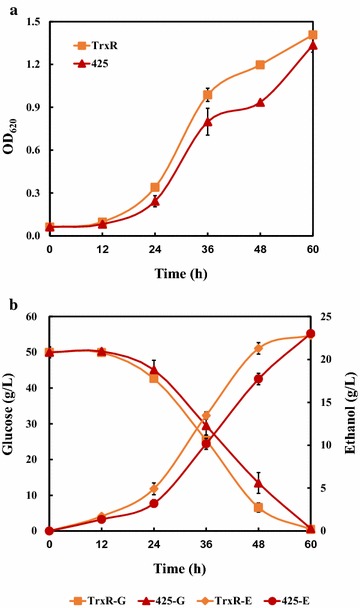
Fermentation profile in *KmTrxR*-expressing *S. cerevisiae* cells during batch ethanol production process under FAF stress. **a** Growth behavior of two strains within 60 h. **b** Glucose consumption and ethanol production under FAF stress. Cells were pre-cultured in SC-Leu medium containing 1 mM H_2_O_2_ for 16–18 h. 1% of seed culture was inoculated into a 250 mL flask with a 100 mL working volume. Data are given as mean ± SD, n = 4

Batch fermentation was conducted in flask levels containing 50 g/L of glucose and FAF inhibitors (0.3 g/L of formic acid, 1.2 g/L of acetic acid, and 0.5 g/L of furfural) to simulate real lignocellulosic hydrolysates. As shown in Fig. 4 and Table 1, the over-expression of *KmTrxR* gene in cells helped to accelerate the process of fermentation under inhibitors, and to reduce the lag phase. The greatest differences of these two strains were between 24 and 48 h, when glucose consumption rate and ethanol production rate of TrxR achieved up to 1.50 and 0.68 g/L/h, respectively, both of which were about 15% more than those in control strain. Particularly, there were significant differences in fermentative parameters between two strains, such as residual glucose concentrations, ethanol concentrations, and productivities within 48 h because of an accelerated fermentative process (Table 1), which may make great differences to industrial-scale ethanol production.

**Table 1:** Fermentative performance under FAF inhibitors within 48 h

	TrxR	425	Trx-TrxR	423–425
Lag phase, h	12	12	0	24
Total glucose, g/L	49.93 ± 0.30	50.27 ± 0.23	50.30 ± 1.76	50.30 ± 1.76
Residual glucose, g/L	6.55 ± 1.28	13.43 ± 2.92	12.51 ± 0.38	23.74 ± 1.19
Glucose consumption rate, g/L/h^a^	1.50 ± 0.08	1.32 ± 0.01	1.43 ± 0.02	1.10 ± 0.04
Glycerol, g/L	1.79 ± 0.03	1.55 ± 0.02	1.46 ± 0.00	1.04 ± 0.02
Ethanol, g/L	21.30 ± 0.66	17.73 ± 0.68	14.75 ± 0.74	11.44 ± 0.23
Etahnol generation rate, g/L/h^a^	0.68 ± 0.00	0.60 ± 0.01	0.55 ± 0.01	0.47 ± 0.00
Productivity, g/L/h	0.44 ± 0.01	0.37 ± 0.00	0.31 ± 0.02	0.24 ± 0.00

^a^Data was calculated between 24 and 48 h

### Synergistic effect of *KmTrxR* and *KmTRX* in *S. cerevisiae*

To verify the synergistic effect of *KmTrxR* and *KmTRX* to stress tolerance, the engineered strains with double gene expressions were constructed. Stress tolerance assay as above was subsequently conducted.

**Fig. 5:**
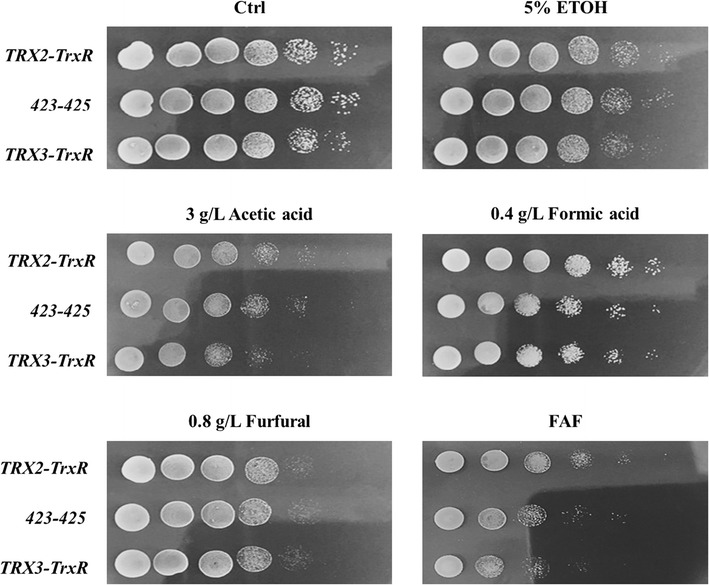
Synergistic effect of *KmTrxR* and *KmTRX* over-expression to multiple inhibitors by serial dilution assay. Cells in log phase with OD_620_ of 10 were serially diluted to 10^−5^, and then spotted onto SC-Leu-His plates containing various inhibitors. Cells were cultivated at 30 °C for 3 days and then photographed

As is shown in Fig. 5, in serial dilution assay, no significant differences in growth were discovered under no inhibitors, of the double expression strains (Trx2-TrxR and Trx3-TrxR) and control strain (423–425). There were no obvious differences in cell growth under conditions of ethanol, acetic acid, and furfural in strains of Trx2-TrxR and 423–425. In particular, the tolerance of Trx2-TrxR strain to formic acid was greatly increased, which was 1–2 gradients more than control strains. More importantly, the enhancement of the tolerance to a mixed FAF inhibitors in overexpressed strain (Trx2-TrxR) was detected, which was also increased by at least 2 gradients. However, the synergistic effect of *KmTRX3* and *KmTrxR* was barely observed under the same conditions (Fig. 5).

**Fig. 6:**
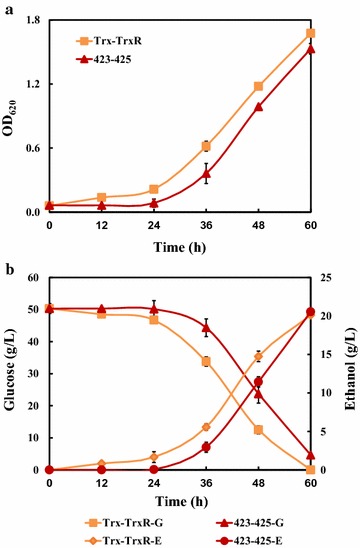
Fermentation profile in Trx2-TrxR strain during batch ethanol production process under FAF stress. **a** Growth behavior of two strains within 60 h. **b** Glucose consumption and ethanol production under FAF stress. Cells were pre-cultured in SC-Leu-His medium containing 1 mM H_2_O_2_ for 16–18 h. 1% of seed culture was inoculated into a 250 mL flask with a 100 mL working volume. Data are given as mean ± SD, n = 4

Furthermore, batch ethanol fermentation was performed in media containing 50 g/L glucose and FAF inhibitors, to further evaluate the possible synergistic effect of *KmTRX2* and *KmTrxR*. Coupled with *KmTrxR* gene, the overexpression of *KmTRX2* gene accelerated the fermentative process, and shortened the lag phase. Strain Trx2-TrxR showed no lag phase, which was superior to those in TrxR and 425. More importantly, the glucose consumption rate of Trx2-TrxR increased by over 30%, achieving 1.43 g/L/h, compared to that of 13% between TrxR and 425 (Fig. 6 and Table 1).

### Mechanisms of an enhanced tolerance related to intracellular ROS levels

**Fig. 7:**
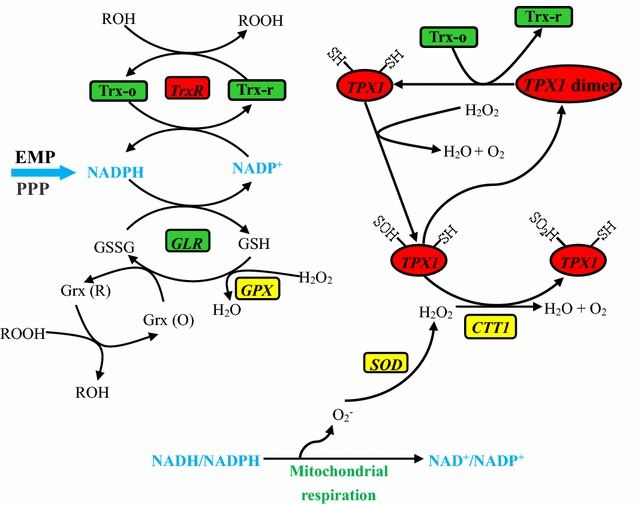
Oxidative defense pathways and essential antioxidant genes in yeast cells

According to existing theories and our results, the possible mechanisms of oxidordeuctases enhancing tolerance to inhibitors is inferred to be related to the levels intracellular ROS. When cells were exposed to inhibitors, ROS like OH·, H_2_O_2_, and O_2_^·−^ might be generated, which may cause molecular damages and cellar effects to the normal cells, and eventually lead to cell death [10]. However, some oxidordeuctases achieved functional dimer proteins after transcription and translation under peroxides [31]. The activated dimers removed excess ROS inside cells to maintain normal cell metabolism and to ensure a high rates of cell viabilities (Fig. 7).

**Fig. 8:**
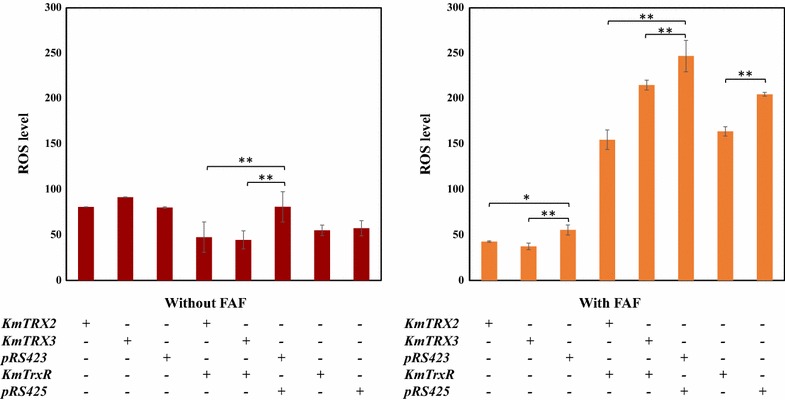
Intracellular ROS levels in recombinant strains with different target genes with, or without FAF inhibitors by DCFH-DA (n = 4, *P < 0.05, **P < 0.01)

Fortunately, the measurement of intracellular ROS provided strong supports for our inference (Fig. 8). Without inhibitors FAF, the levels of intracellular ROS in strains with *KmTRX*, or *KmTrxR* genes almost remained consistent, and the double expression strains (Trx2-TrxR and Trx3-TrxR) contained much lower ROS than the control strain (423–425). However, the very significant differences occurred when cells were exposed to FAF inhibitors. The levels of intracellular ROS in overexpressed strains decreased dramatically, especially strain with both *KmTRX2* and *KmTrxR* with a 40% reduction. Consequently, we concluded that a more effective remove of ROS in over-expressed strains was an important guarantee of growth, metabolism and multiplication of yeasts.

## Discussion

Under certain conditions, ROS generated from cell metabolism causes more cell damages, and ROS generally includes super oxide, peroxide, and hydroxyl radical et al. [32]. In order to prevent from ROS damages, microorganisms build a perfect defense system, which involves some essential oxidoreductases and compounds, such as peroxiredoxins (Prxs), thioredoxin (*TRX*) and its reductase (*TrxR*), glutathione and its reductase (*GLR*) and peroxidase (*GPX*), catalase (*CTT1*), and superoxide dismutase (*SOD*) (Fig. 7a). These oxidoreductases might be also related to the tolerance to some specific inhibitors [32, 33]. Actually, the peroxiredoxin, encoded by *KmTPX1*, has been proved to contribute to an obvious enhancement of tolerance to both oxidative stress and lignocellulose-derived inhibitors [29]. Therefore, to further explore the potential functions of oxidoreductases on cells survival under tough conditions, the thioredoxin system from *K. marxianus*, composed of thioredoxin (*KmTrx*) and its reductase (*KmTrxR*), was selected as the potential targets to increase the stress tolerance in yeasts [28].

The thioredoxin system is widely present in most yeast mitochondria and acts as a defense against ROS damages. Thioredoxins are thiol-disulfide oxidoreductases that involve in a variety of cellular functions depending on its conserved active sites WCXXC [34, 35]. Both thioredoxin genes detected in *K. marxianus* (*KmTRX2* and *KmTRX3*) possess the conserved domain (–CGPC–, Fig. 1d; Additional file 1: Figure S2). In spite of the shortest distance with the *TRX1* gene in *S. cerevisiae* (Fig. 1c), *KmTRX2* was defined as previously described [27]. Active thioredoxin system requires that thioredoxins are maintained at reduced form by its reductase (Fig. 7a). Thioredoxin reductase, as a kind of flavoenzyme, also contains one or two dithiol-disulfide motifs [36]. Except for the typical dithiol-disulfide motifs, two additional cysteine residues may possess their potential structural and functional roles in *KmTrxR* (Fig. 1b; Additional file 1: Figure S1), considering that TrxR catalyzes reactions using NADPH via FAD molecular and the redox-active cysteine residues [37].

Trx-TrxR system in Archaea, bacteria, plants, and lower eukaryotes (such as yeasts) contributes to regulating the intracellular redox balance [16, 30, 34, 35] In particular, a thioredoxin from *Endocarpon pusillum* showed its distinctive role in stress tolerance [34], which strikes us that the gene *KmTRX* and *KmTrxR* may possess the potential applications in increasing tolerance of yeasts to some specific inhibitors during ethanol fermentation. To test the possible functions of *KmTrxR* and *KmTRX2/3*, the preculture of yeast cells were incubated with low concentration of peroxide (1 mM) to turn on the specific reactions, and the possible influence of indigenous redox system in *S. cerevisiae* by the addition of H_2_O_2_ may be excluded by our experimental settings based on the same background, and by the facts that similar growth behaviors were achieved among all strains without inhibitors (Figs. 2, 3, 5). In this case, our results indicated that single overexpression of both Trx genes from *K. marxianus* showed a weak association with an enhanced stress tolerance (Fig. 2). Actually, the repressive effect of *KmTRX3* in tolerance to formate makes it distinguished from other Trxs in cells. Compared to Trx1 and Trx2, specific location and sequence of Trx3 in yeast was reported [38], which might be related to the negative responses. In this case, the possible functions of gene *KmTrxR* and the synergistic effect of *KmTRX* and *KmTrxR* were further explored by serial dilution assay and batch ethanol fermentation.

On the one hand, single expression of gene *KmTrxR* was achieved in *S. cerevisiae* cells (Figs. 3, 4). First, considering lignocellulose-derived inhibitors, inducing the generation of intracellular ROS that is closely related to the redox state in cells [10–12], an enhanced tolerance to multiple lignocellulose-derived inhibitors, especially to formic acid and acetic acid, were observed in *KmTrxR*-overexpressing strain by serial dilution assay (Fig. 3). These observations have also been supported by a variety of similar oxidoreductases that might be contributed to the increased tolerance of *S. cerevisiae* to the inhibitors [17, 19–23, 39]; Besides, different from previous results achieved, such as a 2-Cys Prx from *Oryza sativa* [39] and a thioredoxin from *Endocarpon pusillum* [347], the principal advantage of *KmTrxR* gene tends to increase a comprehensively enhanced tolerance of yeasts to a mixture of lignocellulose-derived inhibitors (FAF), which may provide more references and potential applications for ethanol production from lignocellulosic materials (Fig. 3). Surprisingly, an obviously enhanced tolerance of strain TrxR to high concentration of salt was detected (data not shown). These finding is supposed to validate our previous inferences, and makes *KmTrxR* a promising gene in ethanol production from lignocellulosic materials. Finally, a better fermentative profile was achieved when cells were exposed to FAF mixtures (Fig. 4) during batch ethanol production, which was manifested as the shorter lag period and accelerated process in the fermentation, just as expected previously [18, 39].

On the other hand, the possible synergistic effect of *KmTRX* and *KmTrxR* was evaluated to strengthen the functions by overexpressing single *KmTrxR* gene (Figs. 5, 6). In spite of the contribution to an enhanced stress tolerance of *KmTrxR* gene, the poor effect, compared to the previous *KmTPX1* gene [29] makes little sense. Fortunately, the double overexpression strain (Trx2-TrxR) showed the greatly enhanced tolerance to the mixed FAF inhibitors, especially for the formic acid among it (Fig. 6). Considering the negative effect on acetic acid and furfural, the enhancement was supposed to be consequences of amplification effect on formic acid.

The reasonable growth behavior under conditions of FAF by the synergistic effect of *KmTRX2* and *KmTrxR* was superior to those by single overexpression of *KmTRX* and *KmTrxR*. The fermentative process of TrxR and 425 (SC-Leu) was much faster than that of Trx2-TrxR and 423–425 (SC-Leu-His), considering the differences in nutrition. Even so, we found that the differences of Trx-TrxR and 423–425 in glucose consumption rate and productivity were very significant (P < 0.01), compared with that were only significant (P < 0.05) between TrxR and 425 (Table 1; Additional file 1: Figure S4). In particular, the Trx2-TrxR strain achieved a greatly enhanced ethanol fermentation profile, compared with that of TrxR strain (Figs. 4, 6, Table 1). The increase of glucose consumption (by 30% vs 13%) with shorter lag phase from strain Trx2-TrxR under nutrition limited conditions (Table 1; Additional file 1: Figure S4), has all proved that *KmTrxR* gene coupled with *KmTRX* gene plays a more significant role in promoting stress tolerance to multiple lignocellulose-derived inhibitors than each single gene. Therefore, these findings under existing conditions further confirmed the synergistic effect of *KmTRX* and *KmTrxR*, which may lay a good foundation for its applications in the future.

In a word, the recombination *S. cerevisiae* with *KmTRX* and *KmTrxR* overexpressing possesses an enhanced tolerance to multiple lignocellulose-derived inhibitors, such as formic acid, acetic acid, and even the FAF mixtures (Figs. 3, 6). However, considering the complicated mechanisms that these inhibitors are toxic to cells, they may, as what we all know, induce cells to generate intracellular ROS [10–12], which has also been proved by our results in Fig. 8. Therefore, oxidoreductases help to remove the excess ROS, maintaining it at a normal level, to achieve an elevated tolerance and to reduce damages to cells. Intracellular ROS in cells with an increased tolerance has been reported to decrease under inhibitors, no matter what strategies were adopted [18, 39, 40].

An obviously decreased levels of intracellular ROS in overexpressed strains have proved that the overexpression of *KmTRX* and *KmTrxR* gene helps cells to maintain a normal amount of ROS (Fig. 8). Single expression of *KmTRX2* and *KmTRX3* contributed little to the reduction of ROS levels, which was in line with their negative functions in tolerance to inhibitors. In spite of a 20% reduction of ROS in *KmTrxR*-overexpressing strain, the promotion on an enhanced tolerance to FAF inhibitors was so limited. On this basis, the double expression of *KmTRX2* and *KmTrxR* played a more positive role in removing the intracellular ROS levels (with an over 40% reduction) with, or without inhibitors, which further confirmed the synergistic effect of these two genes. Above all, our results and some previous reports all confirmed that oxidoreductases maintained a normal level of intracellular ROS, which eventually manifested as an apparently enhanced tolerance of *S. cerevisiae* to multiple lignocellulose-derived inhibitors [18, 39, 40].

## Conclusions

An enhanced tolerance of yeasts to multiple lignocellulose-derived inhibitors contributes to the process of lignocellulosic ethanol production. Here we reported that the overexpression of both thioredoxin and its reductase from *K. marxianus* can enhanced tolerance to multiple lignocellulose-derived inhibitors and achieved the remarkable ethanol fermentative profiles under inhibitors. Actually, compared with single gene expression, the synergistic effect of *KmTRX2* and *KmTrxR* was further confirmed under a mixture of formic acid, acetic acid, and furfural (FAF) with a shorter lag period. At last, the mechanism that improves the tolerance depended on a normal level of intracellular ROS for cell survival under stress. Consequently, all these findings provided the potential possibility for ethanol production from lignocellulosic materials, and give a general insight into the possible toxicity mechanisms for further theoretical research.

## Methods

### Strains, media and growth conditions

All strains used in this study are listed in Table 2. Yeast cells were grown at 30 °C in YPD medium (2% glucose, 1% yeast extract and 2% peptone) or in synthetic complete medium (SC) containing 2% glucose and 0.67% yeast nitrogen base, supplemented with the appropriate amino acids (Additional file 1: Table S1). *Escherichia Coli* DH5α was cultivated at 37 °C in LB medium (0.5% yeast extract 1% peptone and 1% sodium chloride).

**Table 2:** Strains, plasmids and primers used in this study

Strains	Genotype	Source
Y179	*K. marxianus*, wild-type	CCTCC (M202031)
280	*S. cerevisiae* BY4741, *MAT*a, *his3*-*1, leu2*-*0, met 15*-*0, ura3*	ATCC (201388)
DH5α	*E. coli* competent cells for genetic manipulation	Invitrogen (18258012)
TrxR	*S. cerevisiae* over-expressing *KmTrxR* gene, transformated from 280	This study
425	*S. cerevisiae* with pRS425 plasmid, transformated from 280	This study
Trx2	*S. cerevisiae* over-expressing *KmTRX2* gene, transformated from 280	This study
Trx3	*S. cerevisiae* over-expressing *KmTRX3* gene, transformated from 280	This study
423	*S. cerevisiae* with pRS423 plasmid, transformated from 280	This study
Trx2-TrxR	*S. cerevisiae* over-expressing both *KmTRX2* gene and *KmTrxR* gene, transformated from 280	This study
Trx3-TrxR	*S. cerevisiae* over-expressing both *KmTRX3* gene and *KmTrxR* gene, transformated from 280	This study
423–425	*S. cerevisiae* with both pRS423 and pRS425 plasmid, transformated from 280	This study

Plasmids	Characteristic	Source and reference
pRS423	Yeast episomal vector with *HIS3* marker	[41]
pRS425	Yeast episomal vector with *LEU2* marker	[41]
p425TrxR	*KmTrxR* gene in pRS425	This study
p423Trx2	*KmTRX2* gene in pRS423	This study
p423Trx3	*KmTRX3* gene in pRS423	This study

Primers		
KmTrxR-F	5′-TCC*gagctc*CCCATGTCAAATGATGAAACG-3′	
KmTrxR-R	5′-TCC*ccgcgg*ACGAGGAACCAACCTTTATT-3′	
KmTrx2-F	5′-GAGTCC*gagctc*CGGTTTGTTTACCAATCGAT-3′	
KmTrx2-R	5′ ATAGCG*actagt*ACTTCGACTATGCGTTGGCC-3′	
KmTrx3-F	5′-GAGTCC*gagctc*TGCCAAAACTGAACTTCCAT-3′	
KmTrx3-R	5′-ATATAT*actagt*CTTCCTCGGGCAGCTCGGAC-3′	

*CCTCC* China Center for type culture collection, *ATCC* American type culture collection

### Plasmids construction and transformation

To construct an overexpression vector with the *KmTrxR* gene and resistance gene, plasmid pRS425 [41] was used for DNA manipulation and cloning. DNA manipulation was performed by standard procedures [42]. *KmTrxR* gene fragment, amplified from genome of *K. marxianus* Y179 with primers KmTrxR-F and KmTrxR-R. The obtained fragment with a coding region and a native promoter of *KmTrxR* gene was inserted into pRS425 to generate pRS425-TrxR after digestion with *Sac*I and *Sac*II. The constructions were verified by both digestion of restriction enzyme and sequencing. Similarly, *KmTRX2* and *KmTRX3* gene with their own promoters, amplified with the corresponding primers in Table 2, were inserted into plasmid pRS423 with restriction enzymes of *Sac*I and *Spe*I.

Transformation of *E. coli* cells was referred to CaCl_2_ method [43]. For screening the transformants, ampicillin (100 μg/mL) was added into LB selective plates. Yeast transformation was conducted by LiAc/PEG method described by Gietz et al. [44], and transformants were screened by SC medium without leucine (SC-Leu), or Histidine (SC-His), or both (SC-Leu-His). Transformants with the recombinant plasmids were marked as described in Table 2 in this study. All transformants were verified by PCR to prove the presence of the constructs. To test the normal functions of all overexpressed genes under the control their native promoters, the qPCR analysis of all recombinant strains with corresponding target genes were performed (Additional file 1: Tables S2, S3). A detailed description could be found in Additional file 1.

### Stress tolerance assay

To test functions of *KmTrxR* gene and *KmTRX* gene, serial dilution assay were performed using recombinant strains. Yeast cells were cultivated in corresponding selective medium with 1 mM H_2_O_2_ at 30 °C and 150 rpm for 16–18 h (OD_620_ = 2), and then collected by centrifugation at 3000×*g* for 5 min. Concentrated cells (final OD_620_ = 10) were serially diluted with distilled water, after which 10 μL of the diluted cells was loaded onto SC selective agar plates containing a single or mixed lignocellulose-derived inhibitors. The inhibitors applied in this section involved in formic acid, acetic acid, furfural, and their mixture FAF. All plates were incubated for 2–3 days at 30 °C and then photographed. For high-temperature test, plates were incubated at 42 °C.

### Laboratory-scale batch fermentation

To evaluate fermentative performance of strain TrxR and strain Trx2-TrxR, batch fermentations were conducted in a flask level for around 60 h. Pre-cultures of yeast cells were carried out in SC selective medium with 1 mM H_2_O_2_, and then cells was inoculated in corresponding SC medium containing 50 g/L of glucose and FAF mixture (0.3 g/L formic acid, 1.2 g/L acetic acid and 0.5 g/L furfural) at 30 °C and 150 rpm. The initial OD_620_ was adjusted to 0.03, and samples were taken every 12 h to test cell growth, sugar and by-products.

### Bioinformatics analysis

Evolutionary tree of some *KmTRX* genes and *KmTrxR* genes from both Y179 and its allied species using *MEGA 4* software [45], and amino acid sequences applied are listed as follows: *KmTrxR*, thioredoxin reductase from *K. marxianus* Y179 in this study; *KlTrxR*, thioredoxin reductase from *K. lactis* (Accession No. XP_454928.1); *ScTrxR*, thioredoxin reductase from *S. cerevisiae* (Accession No. NP_010640.1); *CtTrxR*, thioredoxin reductase from *Candida tropicalis* (Accession No. XP_002546509.1); *SsTSA1*, thioredoxin reductase from *Scheffersomyces stipitis* (Accession No. XP_001384831.1); *AnTrxR*, thioredoxin reductase from *Aspergillus niger* (Accession No. XP_001389279.1); *EcTrxR*, thioredoxin reductase from *E. coli* (Accession No. ADX51538.1); *EpTrxR*, thioredoxin reductase from *Endocarpon pusillum* (Accession No. AEH41540.1); *KmTRX2* and *KmTRX3*, thioredoxins from Y179; *ScTRX1*, thioredoxin 1 from *S. cerevisiae* (Accession No. NP_013144.1); *ScTRX2*, thioredoxin 2 from *S. cerevisiae* (Accession No. NP_011725.3); *ScTRX3*, thioredoxin 3 from *S. cerevisiae* (Accession No. NC_001135.5); *KlTRX*, thioredoxin from *K. lactis* (Accession No. XP_454686.1); *CtTRX2*, thioredoxin 2 from *C. tropicalis* (Accession No. XP_002546341.1). Alignment was conducted by the National Center for Biotechnology Information (NCBI) Basic Local Alignment Search Tool (BLAST) software (http://clustalw.ddbj.nig.ac.jp/).

The three dimensional (3D) structure of *KmTrxR* was predicted by SWISS-MODEL (http://swissmodel.expasy.org/) and the 3D structure of TrxR from *S. cerevisiae* (PDB Accession No. 3ITJ) was adopted as the structure template. Similarly, the 3D structure template of *KmTRX* gene was also from the homologous *ScTRX2* from *S. cerevisiae* (PDB Accession No. 2hsy.1.A). Software RasMol was used to view and analyze the predicted 3D structure of *KmTrxR*, as was shown in Fig. 1.

Expression pattern analysis of differentially expressed genes related to oxidative stress from transcriptome of *K. marxianus* Y179 [28] was clustered using Cluster software [46] and Java Treeview software [47]. The hierarchical clustering of the chosen experimental conditions and genes was carried out by using Euclidean Distance as the formula of the distance matrix.

### ROS level analysis

Levels of ROS inside cells were tested by a common method using 2′,7′-dichlorofluorescein diacetate (DCFH-DA, Sigma-35845, dissolved in absolute ethanol) as an indicator. Cells after pre-culture were cultivated for 16–18 h in corresponding selective medium with FAF (0.3 g/L formic acid, 1.2 g/L acetic acid and 0.5 g/L furfural) at 30 °C and 150 rpm (OD_620_ 1.0). Cell pellets were washed twice with distilled water, and then re-suspended in 0.5 mL 10 mM PBS (pH 7.0) containing 10 μM DCFH-DA. After incubation at 37 °C for 60 min, fluorescence was measured by a Multiskan spectrum microplate spectrophotometer (PerkinElmer, USA) with λ_EX_ 485 nm and λ_EM_ 525 nm [40].

### Analytical methods

The cell concentration was measured using the optical density at 620 nm. Concentrations of glucose, ethanol and glycerol were analyzed by Aminex HPX-87H column (300 × 7.8 mm; Bio-Rad, Hercules) in HPLC system, which used 0.01 mol/L H_2_SO_4_ as mobile phase and eluted at 50 °C with a flow rate of 0.5 mL/min. All analysis in this study was done in quadruplicate except indicated, and the mean values are shown in “Results” and “Discussion” section.

## Additional file

**Additional file 1.** Supplemental methods, tables and figures.

## Abbreviations

ROS: reactive oxidative species
*KmTRX*: thioredoxin gene from *K. marxianus*
*KmTrxR*: thioredoxin reductase gene from *K. marxianus*
FAF: a mixed inhibitors of formic acid, acetic acid and furfural
SC: synthetic complete medium
SC-Leu: SC medium without Leucine
SC-Leu-His: SC medium without Leucine and Histidine
DCFH-DA: 2′,7′-dichlorofluorescein diacetate
