# Supplementary material for: Synergistic effect of thioredoxin and its reductase from Kluyveromyces marxianus on enhanced tolerance to multiple lignocellulose-derived inhibitors
Source: Microb Cell Fact. 2017 Oct 30;16:181. doi: 10.1186/s12934-017-0795-5 (PMC5663110; doi:10.1186/s12934-017-0795-5)
Supplement: Supplementary file 1 — Additional file 1. Supplemental methods, tables and figures. [file 12934_2017_795_MOESM1_ESM.docx]

**Additional materials for *Microbial Cell Factories***

**Synergistic effect of** **thioredoxin and its reductase from *Kluyveromyces marxianus* on enhanced tolerance to multiple** **lignocellulose-derived inhibitors**

Jiaoqi Gao^1^, Wenjie Yuan^1*^, Yimin Li^1^, Fengwu Bai^2^, Yu Jiang^3^

^1^School of Life Science and Biotechnology, Dalian University of Technology, Dalian 116024, China

^2^State Key Laboratory of Microbial Metabolism, Shanghai Jiaotong University, Shanghai 200240, China.

^3^Department of Pharmacology and Chemical Biology, University of Pittsburgh, PA, 15261, USA.

^*^**Corresponding author:** Dr. Wenjie Yuan,

Tel: +86-41184706329 Fax: +86-41184706329

E-mail address: ywj@dlut.edu.cn;

**Methods**

**Real-time quantitative PCR**

Real-time quantitative PCR (qPCR) were performed to verify the expression levels of the *KmTRX2* gene, *KmTRX3* gene, and *KmTrxR* gene under its native promoter. Samples were taken at log phase. Cell pellets were collected by centrifugation at 5000 g at 4°C for 5 min, and were then frozen by liquid nitrogen. The total RNA of every sample was extracted by the RNeasy® Mini Kit (Qiagen, Hilden, Germany) according to the manufacturer’s instructions. The procedure of reverse transcription and real-time PCR reaction were described previously [21] using PrimeScript^®^ RT reagent Kit and SYBR^®^ Premix Ex TaqTM II (Takara Bio Inc.), respectively. Primers used in the reactions were listed in Table S2. The data analysis selected actin gene as the endogenous reference gene, and the fold-change of a specific gene was determined by the method of 2^-∆∆C^_T_. Results were normalized by the relative mRNA expression level, which was defined as the fold-change when the expression level of control strain with the empty plasmids was set as 1. Quadruplicate experiments were performed to guarantee the reproducibility of all the results.

Table S1 Composition of amino acids in synthetic complete medium (SC)

| Amino acids | Final concentrations (mg/L) |
| --- | --- |
| Arginine | 20 |
| Aspartic acid | 100 |
| Glutamic acid | 100 |
| Isoleucine | 30 |
| Lysine | 30 |
| Valine | 150 |
| Phenylalanine | 50 |
| Serine | 375 |
| Tyrosine | 30 |
| Threonine | 20 |
| Adenine | 40 |
| Histidine | 20 |
| Leucine | 60 |
| Tryptophan | 40 |
| Uracil | 20 |
| Methionine | 20 |

Table S2 Primers used for real-time quantitative PCR in this study.

| Name | Sequences (5’~3’) |
| --- | --- |
| Trx2-F | CTTCCGCTGCTGATTTCG |
| Trx2-R | ACCTCCTTGCCACCCTTG |
| Trx3-F | ATCTTCCACCAGTAGTAGTTCC |
| Trx3-R | TCATCCACATCCACCTTG |
| TrxR-F | GCGTGCCTCTCAAATCATG |
| TrxR-R | AACGTAGCCAGCATCGTCAA |
| ACT1-F | GCCGAAAGAATGCAAAAGGA |
| ACT1-R | GGAAGGTAGTCAAAGAAGCCAAGA |

Table S3 Relative expression levels of target genes by real-time quantitative PCR

| Strain | Gene | Expression level |
| --- | --- | --- |
| Trx2 | *KmTRX2* | 123 |
| Trx3 | *KmTRX3* | 1384 |
| 423 | *KmTRX2/ KmTRX3* | 1 |
| TrxR | *KmTrxR* | 9286 |
| 425 | *KmTrxR* | 1 |
| Trx2-TrxR | *KmTRX2* | 1178 |
| Trx3-TrxR | *KmTRX3* | 32300667 |
| 423-425 | *KmTRX2/ KmTRX3* | 1 |
| Trx2-TrxR | *KmTrxR* | 229045 |
| Trx3-TrxR | *KmTrxR* | 421485 |
| 423-425 | *KmTrxR* | 1 |

**Figure S1 Alignment of amino acid sequences of thioredoxin reductase from different microorganisms indicates some active domains and sites.**

**Figure S2 Alignment of amino acid sequences of thioredoxin from different microorganisms indicates some active domains and sites.**

**Figure S3 Statistic analysis (T-test) of fermentative parameters from ethanol production under FAF. Data is derived from Table 1, n=4, *P<0.05, **<0.01. GCR, glucose consumption rate, g/L/h; EGR, etahnol generation rate, g/L/h; Qp, productivity, g/L/h.**
